# Effects of Glyphosate on Female Reproductive Output in the Marine Polychaete Worm *Ophryotrocha diadema*

**DOI:** 10.3390/toxics11060501

**Published:** 2023-06-02

**Authors:** Dáša Schleicherová, Marino Prearo, Crystal Di Nunno, Alfredo Santovito

**Affiliations:** 1 Department of Life Sciences and Systems Biology, University of Turin, Via Accademia Albertina 13, 10124 Torino, Italy; crystal.dinunno@edu.unito.it (C.D.N.); alfredo.santovito@unito.it (A.S.); 2 IZS PLV, Istituto Zooprofilattico Sperimentale del Piemonte, Via Bologna 148, 10154 Torino, Italy; marino.prearo@izsto.it

**Keywords:** sex allocation, *Ophryotrocha diadema*, glyphosate, female investment, growth rate

## Abstract

Glyphosate is a broad-spectrum herbicide widely employed in agriculture. Exposure to this genotoxic and endocrine-disrupting compound has adverse effects on terrestrial and aquatic organisms and on humans as well. Here, we explored the effects of glyphosate on female reproductive output and somatic growth rate in the marine polychaete worm, *Ophryotrocha diadema.* Adult focal individuals were exposed to different concentrations of pure glyphosate (0.0, 0.125 0.250, 0.500, 1.000 µg/mL) administered once a week for 3 weeks. Toxic effects and mortalities were observed at the three higher concentrations, whereas only a decrease in growth rate was noted after exposure to 0.125 µg/mL, which did not affect female allocation. An area of focus in future studies should be the effects of contaminants, their metabolites, and ecologically relevant human-driven stressors in the context of global warming.

## 1. Introduction

The global decline of certain aquatic and marine species has been attributed to a wide variety of environmental stressors, including habitat loss and fragmentation, exposure to ultraviolet radiation, virulent pathogens, pollutants, and climate change [1,2]. Anthropogenic pollution has become a leading cause of diversity loss. Both plastic production and herbicide use have continued to increase, with millions of tons of chemical compounds, including glyphosate, accumulating in the environment. Many have adverse effects on the biome and pose human health risks, while others have been reported to mimic or inhibit hormone action and alter normal function of the endocrine system in invertebrates and mammals [3].

Glyphosate, the active ingredient in Roundup^®^ Regular, is a broad-spectrum herbicide for commercial and home use. It is employed in agriculture for weed control, in urban areas for vegetation control, and during harvesting as a crop desiccant [4]. Its harmful effects on certain invertebrate and vertebrate species are known: reductions of earthworm biomass and soil microbial diversity [5]; declines in phytoplankton and zooplankton abundance [6]; toxic effects on chironomid larvae, amphibians, and fish [7,8,9]; and spinal malformations in juvenile fish [10]. For example, low-dose glyphosate was found to alter defence against infection in glyphosate-exposed fish [11,12] and reduce cytokine expression in carp [13].

Moreover, toxic effects of exposure to glyphosate have also been observed in mammals and humans and the nervous system of mice [14]. Recent studies have reported its endocrine-disrupting activity in humans [15,16,17,18]. Kwiatkowska et al. [19] found that exposure to glyphosate can induce DNA damage in human leukocytes, resulting in epigenetic alterations in animal/human cells. Glyphosate exposure has been associated with an increased risk of developing Parkinson’s disease and autism [20]. Finally, Santovito et al. [21] analysed the in vitro clastogenic and/or aneugenic effects of glyphosate exposure by studying chromosomal aberrations and micronuclei assays. In detail, exposure of human lymphocytes to five glyphosate concentrations (0.500, 0.100, 0.050, 0.025, 0.0125 μg/mL) resulted in chromosomal aberrations and increased micronuclei frequencies at the higher concentrations. Their study provided evidence for the cytogenetic effects of glyphosate on cultured human lymphocytes. In brief, glyphosate may be considered an endocrine disruptor as well as a genotoxic and neurotoxic carcinogen.

There exists some discrepancy between field observations and experimental ecological research on the harmful impacts of environmental xenobiotics, including glyphosate, on aquatic and marine ecosystems. Published data for the effects of glyphosate on aquatic invertebrates are scarce, particularly for polychaete species [7,22,23]. Amid et al. [23] evaluated the effects of glyphosate exposure and increased temperature in the tropical coral *Acropora formosa* and found loss of colour and chlorophyll. Their findings suggest that coral bleaching can result from different stressors occurring concomitantly, including a toxic effect of glyphosate. In one study [22] in which a polychaete species was used to test glyphosate toxicity, adult individuals of the estuarine polychaeta *Laeonereis acuta* were exposed to various glyphosate concentrations. Alterations in parameters demonstrated the toxic effects of glyphosate on *L. acuta*.

The effects of glyphosate exposure on aquatic and marine invertebrates are still poorly understood. Research into evolutionary ecological changes in invertebrate systems in response to anthropogenic pollution is needed. To fill this gap, we explored the effects of glyphosate exposure on female reproductive output and somatic growth rate in *Ophryotrocha diadema,* a polychaete worm and common species of marine zooplankton. 

## 2. Materials and Methods

### 2.1. Study Model

*Ophryotrocha diadema* (Dorvilleidae) is a simultaneous hermaphrodite with external fertilization. The data on the life cycle of this 1 mm long marine polychaete worm [24,25] and its mating system [26,27,28,29,30,31] have been obtained through laboratory observation. The life cycle consists of a protandrous phase, followed by a simultaneously hermaphroditic phase, which starts when individuals grow to 14 to 15 segments [25] and lasts for about 30 to 40 days. Mating, preceded by 1 to 2 days of courtship, is achieved by pseudocopulation and external fertilization. During external fertilization, the pair maintains close physical contact before releasing their gametes (as described for *O. gracilis* in [32]). The sperm are immotile [33]. The reproductive strategy of *O. diadema* is termed *egg trading*, where partners regularly alternate the male and the female sexual role by reciprocal and conditional exchange of a transparent cocoon containing about 30 eggs every 2 to 3 days. Egg development can be easily followed under a stereomicroscope, since the cocoons are transparent. Nine days after the eggs are laid, the offspring hatch as small four-segment larvae, which can produce viable sperm. The worms become simultaneous hermaphrodites when they reach 14 segments in length [24]. The maximum body length and lifespan of *O. diadema* are 20–21 segments and 90 days, respectively. Two different phenotypes are distinguished, namely, yellow, with yellow oocytes (YY, Yy) and white, with white oocytes (yy). *O. diadema* populations belong to marine zooplankton species that inhabit the organic sediments of fouling fauna of Californian harbours. Though the population density is low, the adults produce a network of mucous trails, so they can be easily followed by conspecifics, which likely makes for a clustered spatial distribution [24]. 

### 2.2. Experimental Set Up

For the present study, we used a liquid solution of glyphosate Roundup^®^ POWER 2.0 PFnPE (Monsanto Agricoltura Italia S.p.A.; Bayer Agriculture BVBA, Antwerp, Belgium) composed of the pure acid glyphosate (360 g/L) in the form of potassium salt (441 g/L), inert substances, and adjuvants. The study was carried out in 10 mL glass bowls filled with artificial sea water, placed in closed boxes to limit evaporation, and kept at 20 °C in a thermostatic cabinet. The animals were fed frozen spinach ad libitum once a week. In order to test the effects of glyphosate exposure on female reproductive output and growth rate, pairs of adult virgin hermaphrodites of the same age and no siblings (offspring (F1) of 24 *O. diadema* parent pairs (PP)) randomly formed five groups (Table 1): 

Control group A. Two adult individuals of *O. diadema*/bowl, 1Yy—focal individual with yellow phenotype and 1yy with white phenotype, in pure artificial sea water.

Experimental group B. Two adult individuals of *O. diadema*/bowl, 1Yy, in pure artificial sea water and exposed to 0.125 μg/mL of glyphosate.

Experimental group C. Two adult individuals of *O. diadema*/bowl, 1Yy and 1yy, in pure artificial sea water and exposed to 0.250 μg/mL of glyphosate.

Experimental group D. Two adult individuals of *O. diadema*/bowl, 1Yy and 1yy, in pure artificial sea water and exposed to 0.500 μg/mL of glyphosate.

Experimental group E. Two adult individuals of *O. diadema*/bowl, 1Yy and 1yy, in pure artificial sea water and exposed to 1.000 μg/mL of glyphosate.

The acceptable daily intake (ADI) established by the European Food Safety Authority (EFSA) for glyphosate is 0.500 μg/mL, whereas the other three concentrations (0.125, 0.250, 1.000 μg/mL) were tested to evaluate the toxicity of glyphosate in a wider range. The animals were exposed to glyphosate administered once a week for 3 weeks, simulating chronic exposure.

For this study, 24 replicates/group were performed, and a total of 144 adult individuals (72 with yellow phenotype: *O. diadema* Yy denotes focals and 72 with white phenotype: *O. diadema* yy) was used. Female reproductive parameters were defined as the number of cocoons laid and number of eggs/cocoon, while the somatic growth rate was defined as the number of setigerous segments. During the 21-day study period, all parameters were measured twice a week. The female reproductive output and the body size of individuals was measured only in focal individuals (*O. diadema* Yy with yellow phenotype). 

### 2.3. Statistical Analysis

Descriptive statistics are reported as means ± standard deviation (SD). The distribution of number of cocoons, eggs/individual, and growth rate was tested for normality using the Kolmogorov–Smirnov one-sample test. Since the distribution of dependent variables was not normal, the non-parametric Kruskal–Wallis test was used to check whether *O. diadema* focal individuals responded differentially to treatment. The number of cocoons, the number of eggs per individual, and the growth rate were dependent variables, and treatment (glyphosate at five different concentrations) was the fixed factor. Statistical significance was set at *p* < 0.05. Data analysis was performed only on focal worms alive at the end of the 3-week study period. Statistical analysis was performed using IBM SPSS Statistics version 27 for Windows (IBM SPSS, Armonk, NY, USA).

## 3. Results

Only data from focal worms alive at the end of the 3-week study period were included in the statistical analysis, while worms from groups C, D, and E were excluded (Table S2). All the individuals in group A (controls) were alive at 3 weeks (24/24 alive, mortality 0%, Table S1). The mortality recorded for group B was 12.5% (21/24 alive, Table S1). The mortality recorded for group C was 91.7% (2/24 alive). The mortality recorded for groups D and E was 100% (0/24 alive, Table S2). 

### 3.1. Effect of Glyphosate Exposure on Female Allocation in O. diadema Focals

The mean number of cocoons was 3.71 ± 0.91 laid by focals in group A and 3.24 ± 0.94 by group B. The mean number of eggs/cocoon laid by focals in group A and in group B was 68.33 ± 23.18 and 56.38 ± 20.65, respectively (Kolmogorov–Smirnov test). 

**Figure 1:**
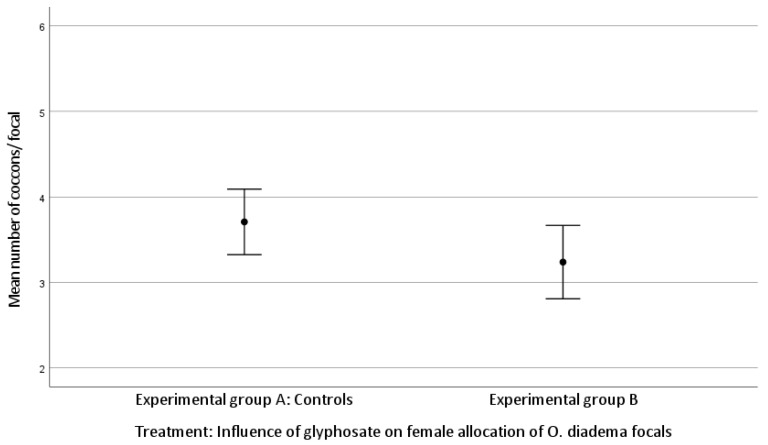
Effect of glyphosate exposure on female allocation (mean number of cocoons/focal) of *O. diadema* focals in group A (controls) and group B (0.125 μg/mL of glyphosate) (*p* > 0.05).

**Figure 2:**
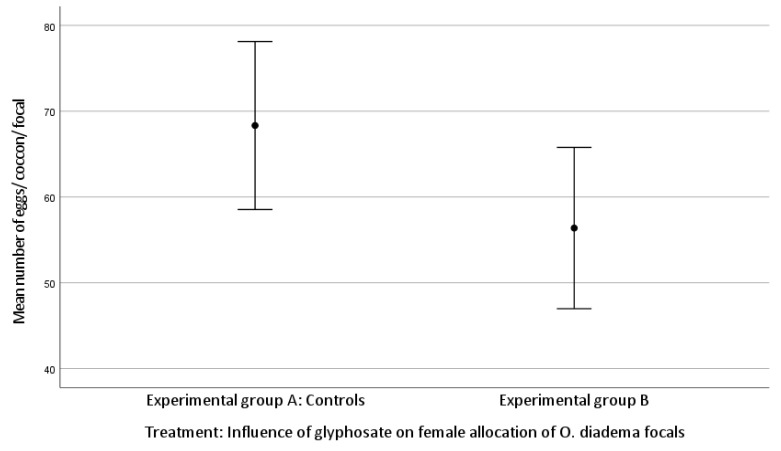
Effect of glyphosate exposure on female allocation (mean number of eggs/cocoon/focal) of *O. diadema* focals in group A (controls) and group B (0.125 μg/mL of glyphosate) (*p* > 0.05).

There was no statistically significant difference in female allocation between group A and group B (Kruskal–Wallis H test: (mean number of cocoons, df 1, *p* = 0.060; mean number of eggs/cocoon, df 1, *p* = 0.057; Figure 1 and Figure 2)). Both *p*-values approached significance and therefore merit attention.

### 3.2. Effect of Glyphosate Exposure on Growth Rate in O. diadema Focals

The mean body growth was 3.79 ± 1.67 setigerous segments in group A and 2.05 ± 1.20 setigerous segments in group B.

**Figure 3:**
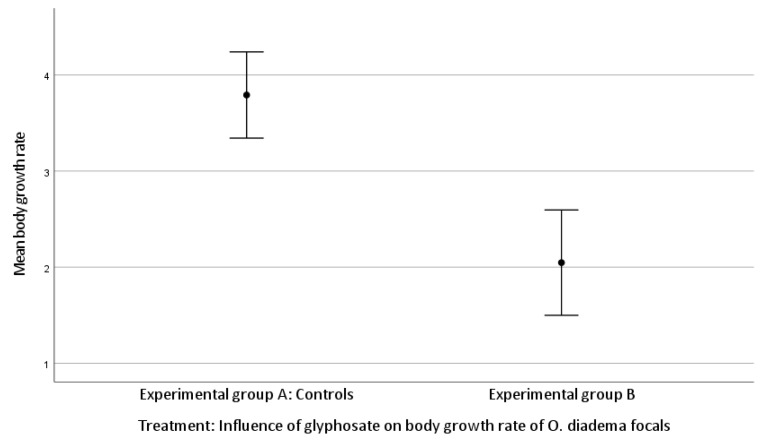
Effect of glyphosate exposure on body growth rate of *O. diadema* focals in group A and group B (*p* < 0.001).

There was a significant difference in body growth rate between group A and group B (Kruskal–Wallis H test, growth rate df 1, *p* < 0.001; Figure 3). 

## 4. Discussion

Glyphosate is the most widely used herbicide in the world. A non-selective systemic biocide with broad-spectrum activity, it is used intensively, with repercussions on environmental health [34]. In their systematic review of the toxic effects of glyphosate on the nervous systems of various animal species and humans, Ferreira et al. [34] reviewed several studies that reported the effects of glyphosate on the central and the peripheral nervous systems of rodents, mainly observed in the development of the nervous system and behavioural changes [35,36]. 

Compared to the numerous studies on glyphosate neurotoxicity in vertebrates, recent studies involving invertebrates are scarce. Two studies reported the neurotoxicity of glyphosate linked to neuronal development, mitochondrial damage, oxidative stress, and behavioural patterns in the hermaphroditic worm *Caenorhabditis elegans* [37,38]. The toxic effects of glyphosate on the endocrine system [39] in marine species are understudied. Uren Webster et al. [40] investigated the effects of glyphosate on reproduction in the zebrafish *Danio rerio* by exposing adult individuals to three different concentrations (0.01, 0.5, 10 mg/L) for 21 days. They found that at the highest concentration, egg production was reduced, and early-stage embryo mortalities and premature hatching were increased, indicating endocrine disruption after exposure to glyphosate. 

Here we investigated the effects of increasing concentrations of glyphosate on female reproductive output in the marine polychaete worm *Ophryotrocha diadema*. We hypothesized that its harmful effects could also influence life-history traits of focals, and we tested its effects on somatic growth rate. Exposure to the two highest concentrations of glyphosate led to mortality in both groups. Harmful effects were observed also in group C; only 8.3% of focals were alive at the end of the 3-week study period. 

We found no significant difference between group A and group B for the number of cocoons laid by focals (*p* = 0.060) or the number of eggs/cocoon (*p* = 0.057). However, since both *p*-values approached significance and the level is a convention, they merit attention. Unlike the effect on female allocation, the somatic growth rate was strongly affected by glyphosate exposure, with a reduced growth rate observed for group B compared to group A. Our data show that the three highest concentrations of glyphosate strongly impacted the health status and the survival of focals, whereas exposure of glyphosate (0.125 μg/mL) affected only body growth rate but not female allocation. These observations are shared by Reddy et al. [41]. In their study, adult individuals of *Lymnea palustris*, an aquatic pond snail, were chronically exposed to ecologically relevant concentrations of glyphosate. Exposure to glyphosate caused alterations in reproduction, growth, and development, as well as reduced fecundity, increased mortality, and developmental abnormalities in offspring. In addition, Asnicar et al. [42] provided evidence for the potential ecotoxicity of glyphosate by exposing individuals at the larval stage of the sea urchin *Paracentrotus lividus* to four environmentally relevant concentrations (1, 10, 50, 100 µg/L) and then recording embryo/larval development. They observed severe alterations in larval development and growth and morphological anomalies. Our observation of reduced somatic growth after exposure to glyphosate is shared by Reddy et al. [41] and Asnicar et al. [42]. In our study, the three highest concentrations were indubitably lethal, whereas exposure to a lower concentration (0.125 μg/mL) was not associated with mortality or reduced fecundity but rather with developmental anomalies. This concentration is very similar to the 100 μg/L Asnicar et al. used [42] and led to the same result.

A future area of focus will be to determine the glyphosate concentration, in a range between 0.125 and 0.250 μg/mL, that has an effect on both somatic growth and female allocation of *O. diadema* focals. Since exposure to 0.125 μg/mL of glyphosate did not have a significant effect on female allocation (i.e., mean number of cocoons and mean number of eggs/cocoon) but only approached statistical significance, we hypothesize that exposure to 0.250 μg/mL may affect somatic growth rate and female allocation.

## 5. Conclusions

In summary, our results show that exposure to glyphosate can induce adverse effects on somatic development (somatic growth rate) in the marine polychaete worm *O. diadema*. Considering that zooplankton form the base of the food chain, our observed findings raise further concerns about glyphosate in marine ecosystems.

## Figures and Tables

**Table 1:** Experimental groups.

Group	Treatment: [Glyphosate]	No. of ind./bowl	No. of Replicates
Control group A	0.0 μg/mL Pure artificial sea water	2 (**1Yy − focal** + 1yy)	24
Group B	0.125 μg/mL	2 (**1Yy − focal** + 1yy)	24
Group C	0.250 μg/mL	2 (**1Yy − focal** + 1yy)	24
Group D	0.500 μg/mL	2 (**1Yy − focal** + 1yy)	24
Group E	1.000 μg/mL	2 (**1Yy − focal** + 1yy)	24

## Data Availability

Not applicable.

## Supplementary Materials

The following supporting information can be downloaded at: https://www.mdpi.com/article/10.3390/toxics11060501/s1, Table S1: Total number of cocoons, total number of eggs/ cocoon and growth rate of *O. diadema* focals during the 21 days of the experimental period in the Control group A and the Experimental group B.; Table S2: Total number of cocoons, total number of eggs/ cocoon and growth rate of O. diadema focals during the 21 days of the experimental period in the Experimental groups D, C and E.
