# Supplementary material for: Effects of Glyphosate on Female Reproductive Output in the Marine Polychaete Worm Ophryotrocha diadema
Source: Toxics. 2023 Jun 2;11(6):501. doi: 10.3390/toxics11060501 (PMC10304119; doi:10.3390/toxics11060501)
Supplement: Supplementary file 1 [file toxics-11-00501-s001.zip › toxics-2409661-supplementary.pdf]

SUPPLEMENTARY MATERIAL:

Table S1. Total number of cocoons, total number of eggs/ cocoon and growth rate of *O. diadema* focals during the 21 days of the experimental period in the Control group A and the Experimental group B.

Control group A<br>gly = 0.0 µg/mL	N. of cocoons	N. of eggs/cocoon	Growth rate	Exp. group B<br>gly = 0.125 µg/mL	N. of cocoons	N. of eggs/cocoon	Growth rate
1	4	67	3	1	4	53	2
2	5	79	3	2	4	65	3
3	5	101	4	3	2	34	2
4	4	90	4	4	3	74	0
5	4	92	3	5	**0**	**0**	**focal died**
6	3	56	4	6	3	67	0
7	4	86	4	7	**0**	**0**	**focal died**
8	3	63	4	8	2	36	1
9	1	10	3	9	4	75	3
10	3	39	4	10	**0**	**0**	**focal died**
11	3	61	4	11	4	76	2
12	4	69	4	12	3	60	3
13	4	93	3	13	4	45	1
14	4	66	4	14	4	67	3
15	4	67	4	15	3	58	0
16	4	59	3	16	3	46	2
17	4	75	4	17	4	79	3
18	4	74	5	18	3	51	4
19	2	20	3	19	4	91	2
20	5	101	4	20	3	63	4
21	3	45	5	21	3	34	3
22	4	59	1	22	5	77	1
23	4	80	4	23	1	9	2
24	4	88	7	24	2	24	2

Table S2. Total number of cocoons, total number of eggs/ cocoon and growth rate of *O. diadema* focals during the 21 days of the experimental period in the Experimental groups D, C and E.

Exp. group C gly = 0.250 µg/mL	N. of cocoons	N. of eggs/cocoon	Growth rate	Exp. group D gly = 0.500 µg/mL	N. of cocoons	N. of eggs/cocoon	Growth rate	Exp. group E gly = 1.000 µg/mL	N. of cocoons	N. of eggs/cocoon	Growth rate
1	**0**	**0**	**focal died**	1	**0**	**0**	**focal died**	1	**0**	**0**	**focal died**
2	**0**	**0**	**focal died**	2	**0**	**0**	**focal died**	2	**0**	**0**	**focal died**
3	**0**	**0**	**focal died**	3	**0**	**0**	**focal died**	3	**0**	**0**	**focal died**
4	**0**	**0**	**focal died**	4	**0**	**0**	**focal died**	4	**0**	**0**	**focal died**
5	**0**	**0**	**focal died**	5	**0**	**0**	**focal died**	5	**0**	**0**	**focal died**
6	**0**	**0**	**focal died**	6	**0**	**0**	**focal died**	6	**0**	**0**	**focal died**
7	**0**	**0**	**focal died**	7	**0**	**0**	**focal died**	7	**0**	**0**	**focal died**
8	**0**	**0**	**focal died**	8	**0**	**0**	**focal died**	8	**0**	**0**	**focal died**
9	**0**	**0**	**focal died**	9	**0**	**0**	**focal died**	9	**0**	**0**	**focal died**
10	**0**	**0**	**focal died**	10	**0**	**0**	**focal died**	10	**0**	**0**	**focal died**
11	**0**	**0**	**focal died**	11	**0**	**0**	**focal died**	11	**0**	**0**	**focal died**
12	**0**	**0**	**focal died**	12	**0**	**0**	**focal died**	12	**0**	**0**	**focal died**
13	**0**	**0**	**focal died**	13	**0**	**0**	**focal died**	13	**0**	**0**	**focal died**
14	3	59	1	14	**0**	**0**	**focal died**	14	**0**	**0**	**focal died**
15	**0**	**0**	**focal died**	15	**0**	**0**	**focal died**	15	**0**	**0**	**focal died**
16	**0**	**0**	**focal died**	16	**0**	**0**	**focal died**	16	**0**	**0**	**focal died**
17	**0**	**0**	**focal died**	17	**0**	**0**	**focal died**	17	**0**	**0**	**focal died**
18	**0**	**0**	**focal died**	18	**0**	**0**	**focal died**	18	**0**	**0**	**focal died**
19	**0**	**0**	**focal died**	19	**0**	**0**	**focal died**	19	**0**	**0**	**focal died**
20	**0**	**0**	**focal died**	20	**0**	**0**	**focal died**	20	**0**	**0**	**focal died**
21	**0**	**0**	**focal died**	21	**0**	**0**	**focal died**	21	**0**	**0**	**focal died**
22	4	80	2	22	**0**	**0**	**focal died**	22	**0**	**0**	**focal died**
23	**0**	**0**	**focal died**	23	**0**	**0**	**focal died**	23	**0**	**0**	**focal died**
24	**0**	**0**	**focal died**	24	**0**	**0**	**focal died**	24	**0**	**0**	**focal died**
